# Idiopathic pulmonary arterial hypertension associated with a novel frameshift mutation in the bone morphogenetic protein receptor II gene and enhanced bone morphogenetic protein signaling: A case report

**DOI:** 10.1097/MD.0000000000017594

**Published:** 2019-10-18

**Authors:** Sun Ha Choi, Youn-Kwan Jung, Ji-Ae Jang, Seungwoo Han

**Affiliations:** a Department of Internal medicine, Kyungpook National University Hospital, Daegu; b Biomedical Research Institute, Gyeongsang National University Hospital, Jinju, Gyeongsangnam-do; c Laboratory for arthritis and bone biology, Fatima Research Institute, Daegu Fatima Hospital, Daegu, Republic of Korea.

**Keywords:** anti-Müllerian hormone receptor type 2, bone morphogenetic protein receptor type II, frameshift mutation, pulmonary arterial hypertension

## Abstract

**Rationale:**

Idiopathic pulmonary arterial hypertension (IPAH) is characterized by intense remodeling of small pulmonary arteries. Loss-of-function mutation of bone morphogenetic protein receptor II (*BMPR2*) gene and exaggerated activation of transforming growth factor (TGF)-β signaling play a critical role in this process.

**Patient concerns and diagnosis:**

We report a novel frameshift mutation (c.117InsT, p.Y40fsX48) of the *BMPR2* gene identified in a 19-year-old IPAH patient with syncope. Despite *BMPR2* mutation, the phosphorylation of Smad2/3 and Samd1/5/8 was increased in the patient's peripheral blood mononuclear cells, and this event was accompanied by the upregulation of bone morphogenetic protein (BMP) signaling target genes, but not TGF-β signaling target genes. Moreover, we observed an increased expression of other BMPRs, that is, anti-Mullerian hormone type-2 receptor and the activin receptor-like kinases (ALK) 1, ALK3, and ALK6.

**Interventions and outcomes:**

The patient was prescribed a combination of macitentan, sildenafil, and nifedipine, which successfully controlled her symptom of syncope and normalized N-terminal pro-brain natriuretic peptide level after 3 months of medication.

**Lessons:**

In light of these results, we propose a new pathogenetic mechanism for IPAH, based on enhanced BMP signaling via the functional replacement of mutated BMPR2 by other BMP receptors.

## Abstract 2

Supplemental Digital Content is available in the text

## 1 Introduction

Idiopathic pulmonary arterial hypertension (IPAH) is a progressive disease characterized by a sustained elevation of pulmonary arterial pressure associated with the obliteration of distal pulmonary arteries.^[1]^ The main histopathologic phenomenon is pulmonary arterial remodeling, including intimal fibrosis, medial thickening, and lumen occlusion, all events in which mutations in members of the transforming growth factor (TGF)-β receptor superfamily play a critical role.^[2]^ Among the involved TGF-β receptors, the bone morphogenetic protein receptor type II (BMPR2) and its mutations are particularly relevant to IPAH. BMPR2 mutations have been identified in about 10% to 25% of sporadic IPAH and more than 70% of familial IPAH patients.^[3–5]^ Currently, the most accredited hypothesis is that loss-of-function BMPR2 mutations result in aberrantly increased TGF-β signaling, which is responsible for the pulmonary arterial remodeling.^[6]^ Here, we report a novel frameshift mutation (c.117InsT, p.Y40fsX48) of the *BMPR2* gene, identified in a 19-year-old IPAH patient. We focused on the changes in bone morphogenetic protein (BMP) and TGF-β signaling associated with this BMPR2 mutation and, in particular, on the aberrant expression of type I and II BMP receptors.

## 2 Materials and methods

For the genetic mutation analysis, we sequenced the *BMPR2* gene coding region from peripheral blood mononuclear cells (PBMCs) and analyzed BMP/TGF-β signaling in the PBMCs of the patient and in those of 2 age- and sex-matched controls with no underlying disease. The detailed experimental methods are described in the Supplementary Methods (see Methods, Supplemental Content, which describe the detailed method used in this paper). All participants gave written informed consent for molecular genetic research studies and publication of clinical data. All exons of the *BMPR2* gene were amplified by polymerase chain reaction (PCR) using primer pairs corresponding to each exon (see Table, Supplemental Content, which lists primer sets for exons of *BMPR2* gene and real-time qPCR primers). This study was approved by the institutional review board of Kyungpook National University Hospital (KNUCH2019-08-016).

## 3 Clinical report and results

### 3.1 Clinical report

A 19-year-old Korean female was admitted with recurrent episodes of syncope. She had experienced exertional dyspnea, 3 years before, and syncope, 2 months before admission. The frequency and duration of syncope had been gradually increasing and she lost her consciousness for 15 minutes at admission. On physical examination, there was evidence of right heart failure with elevated jugular venous pressure and mild lower extremity edema. Abnormal laboratory findings included an elevated concentration of plasma N-terminal pro-brain natriuretic peptide (NT-proBNP), that is, 1682 pg/mL (reference range, 0–125), and serum uric acid, 11.9 mg/dL (reference range, 4.0–7.0). Other parameters, including complete blood count, liver function tests, urinalysis, and cardiac enzymes, were within normal limits. Initial electrocardiogram showed a sinus rhythm, increased amplitude of R wave in V1 to V3, and S wave in V4 to V6, and inverted T waves in leads III, aVF, and V1 to V6, with 1st-degree atrioventricular block. The chest radiograph revealed mild enlargement of the central pulmonary arteries with relative paucity of peripheral vessels. Computed tomography angiogram also showed a dilatation of pulmonary arteries, with right ventricular and atrial enlargement. Two-dimensional echocardiogram revealed a hypertrophied and enlarged right ventricle, as well as enlarged right atrium, with a displacement of interventricular septum toward left ventricle. Doppler tracing of peak tricuspid regurgitation velocity (TRV) was 4.22 m/s and the estimated right ventricular systolic pressure was 86 mm Hg, suggesting IPAH. The patient was prescribed a combination of macitentan (10 mg, daily), sildenafil (20 mg, 3 times a day), and nifedipine (40 mg, daily), and reported no additional episodes of syncope. Follow-up NT-proBNP level normalized to 52 pg/mL and peak TRV to 2.54 m/s after 3 months of medication.

### 3.2 Genetic testing

**Figure 1:**
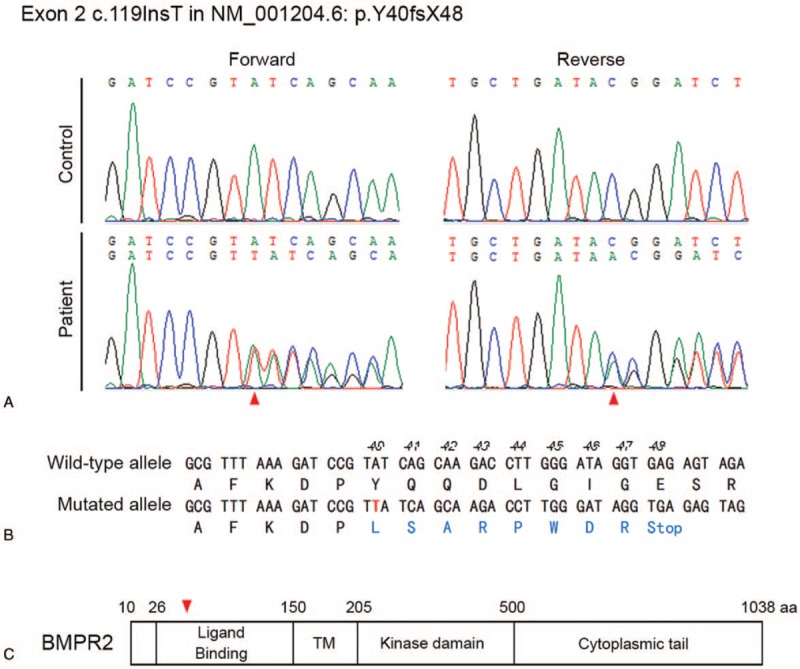
Identification of a frameshift mutation in exon 2 of the *BMPR2* gene. (A) Exon 2 sequencing reveals a heterozygous addition of T at position 117 in the coding sequence, indicated by red arrowhead. (B) This addition made a shift in the grouping of 3 bases, the amino acid changes, and stop codon at amino acid position 48. (C) Schematic structure of the BMPR2 protein. The mutation site indicated by the red arrowhead is located in the extracellular ligand-binding domain. BMPR2 = bone morphogenetic protein receptor II.

DNA sequencing of patient's *BMPR2* gene identified a novel heterozygous frameshift mutation consisting in the insertion of T at the coding nucleotide position 119 (c.119InsT in NM_001204.6) in exon 2 (Fig. 1A). This mutation generates a premature stop codon at a position corresponding to amino acid 48 (p.Y40fsX48: NP_001195.2), which is located in the extracellular ligand-binding domain (Fig. 1B and C), and leads to a truncated BMPR2 protein that does not reach the cell surface.^[7]^ No BMPR2 mutation was detected in the patient's father and sister, who presented no symptoms of pulmonary hypertension.

### 3.3 BMP/TGF-β signaling status and BMPR expression in the patient's PBMCs

**Figure 2:**
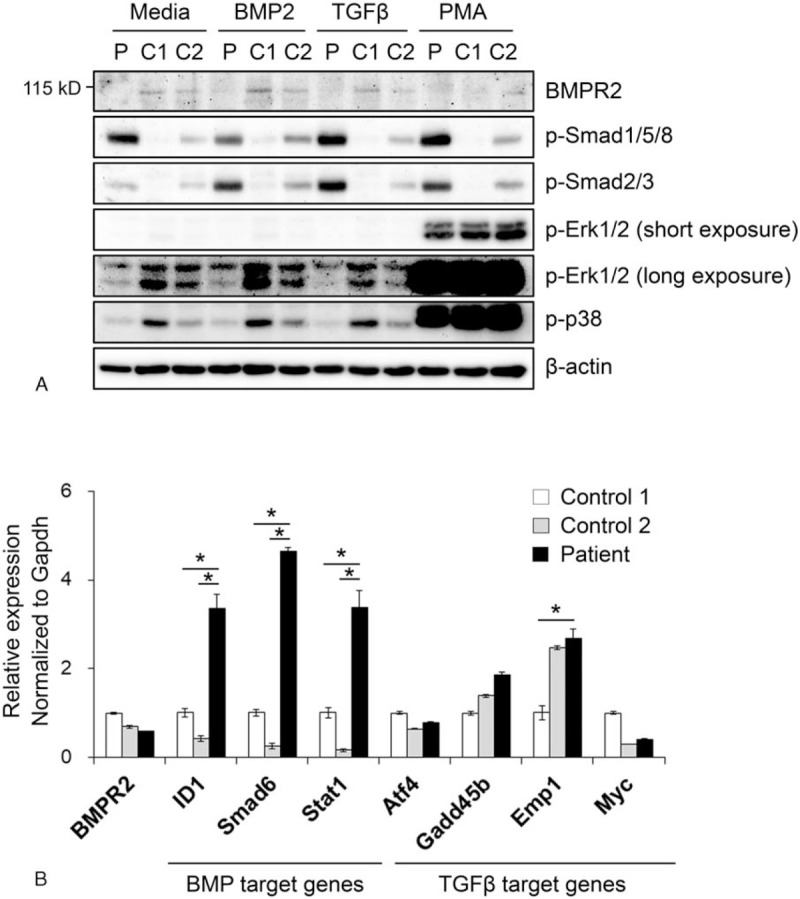
BMPR2 expression and enhanced BMP and TGF-β signaling. (A) Western blot analysis of BMPR2, phosphorylated Smad1/5/8, and Smad2/3, Erk1/2, and p38 MAP kinase. PBMCs were cultured with BMP2 (200 ng/mL), TGF-β (20 ng/mL), and PMA (100 nM) for 24 h to stimulate Smad signaling. C stands for control and P for patient. (B) RNA expression of BMPR2, the BMP target genes, ID1, SMAD6, and STAT1, and the TGF-β-target genes, Atf4, Gadd45b, Emp1, and Myc. PBMCs were cultured in α-MEM-based complete medium for 6 h and harvested for the real-time qPCR. ^∗^*P* < .05 patient versus controls. BMPR2 = bone morphogenetic protein receptor II, PBMCs = peripheral blood mononuclear cells, qPCR = quantitative polymerase chain reaction, TGF-β = transforming growth factor-β.

The protein level of BMPR2 was assessed in the patient's PBMCs and 2 age- and sex-matched controls. PBMCs were cultured in α-MEM-based complete medium with or without BMP2, TGF-β, and PMA, to stimulate Smad and MAP kinase signaling for 24 hours. As expected, the 110 kDa-sized BMPR2 band was almost undetectable in the patient's PBMCs, differently from controls (Fig. 2A). The phosphorylation of Smad1/5/8 and Smad2/3 was substantially increased in the patient compared to controls. On the contrary, the phosphorylation of Erk1/2 and p38 MAP kinase was lower in the patient than in the controls (Fig. 2A). We then assessed the RNA level of BMPR2 and BMP/TGF-β signaling target genes by quantitative RT-PCR. BMPR2 expression in the PBMCs of the patient did not differ from that in the PBMCs of the controls (Fig. 2B). The BMP signaling target genes, ID1, SMAD6, and STAT1, were significantly increased in the patient's PBMCs, whereas the TGF-β target genes, *Atf4*, *Gadd45b*, *Emp1*, and *Myc*, were not (Fig. 2B). These results suggested that an enhanced BMP signaling can be responsible for the pathogenesis of IPAH in this patient.

**Figure 3:**
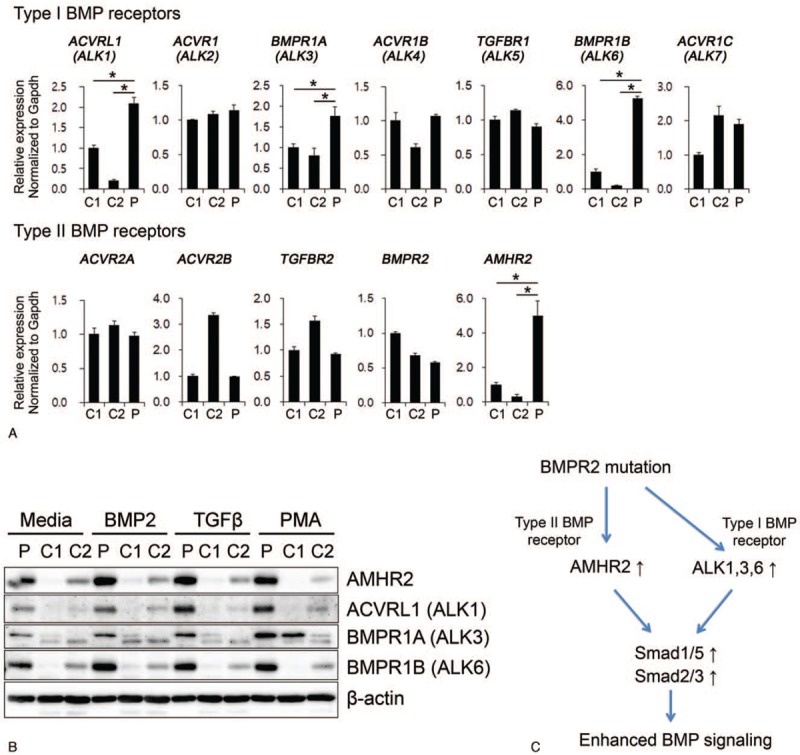
Aberrant expression of BMP receptors and implications for IPAH pathogenesis. (A) RNA expression of type I and II BMP receptors. PBMCs from the IPAH patient and controls were cultured for 6 h in complete medium. (B) Protein level of AMHR2, BMPR1A (ALK3), and BMPR1B (ALK6) in PBMCs treated with growth factors for 24 h. C stands for control and P for patient. (C) Schematic diagram of BMP receptor expression that may result in the enhanced BMP signaling in IPAH. The loss-of-function mutation of BMPR2 is associated with increased expression of other receptors of the TGF-β superfamily, that is, AMHR2 (type II BMPR), as well as ALK1, ALK3, and ALK6 (type I BMPRs), leading to enhanced activation of both Smad1/5/8 and Smad2/3, and increased BMP signaling. ALK = activin receptor-like kinases, AMHR2 = anti-Mullerian hormone receptor 2, BMPR = bone morphogenetic protein receptor, IPAH = idiopathic pulmonary arterial hypertension, PBMCs = peripheral blood mononuclear cells, TGF-β =  = transforming growth factor-β.

The enhanced BMP signaling, in spite of reduced BMPR2, raised the possibility that the function of mutated BMPR2 may be replaced by that of other BMP receptors. Therefore, we examined type I and II BMP receptor expression in PBMCs. In particular, the expression of type I BMP receptors activin receptor like type 1 (ACVRL1, also known as ALK1), BMPR1A (ALK3), and BMPR1B (ALK6) was significantly increased in the patient's PBMCs compared to controls. Among type II BMP receptors, the expression of anti-Mullerian hormone receptor (AMHR) 2 was increased in the patient's PBMCs. This gene exhibited the closest evolutionary relationship with BMPR2 as shown by phylogenetic tree analysis (Fig. 3A).^[8]^ Immunoblotting also demonstrated that the protein level of AMHR2, ACVRL1 (ALK1), BMPR1A (ALK3), and BMPR1B (ALK6) was increased in the patient's PBMCs compared to controls (Fig. 3B).

## 4 Discussion

In this case study, we identified a novel frameshift mutation in exon 2 of the *BMPR2* gene, p.Y40fsX48 (c.119InsT), in a young female patient with IPAH. More than 560 variants have been described for BMPR2 in ClinVar site (https://www.ncbi.nlm.nih.gov/clinvar), and most of the BMPR2 mutations known to be associated with IPAH cause a loss-of-receptor function.^[9,10]^ The c.119InsT mutation generates a premature stop codon at a position corresponding to amino acid 48 in exon 2 and; therefore, a truncated peptide containing only the extracellular ligand-binding domain (Fig. 1). An in-frame deletion in BMPR2 exon 2 was found to prevent BMPR2 expression on the cell surface, causing a retention of the misfolded mutant BMPR2 in the endoplasmic reticulum (ER), and a subsequent increase in ER stress in primary pulmonary endothelial cells.^[7]^

The penetrance of IPAH among individuals with a BMPR2 mutation is only about 20% and is 3 times higher in females than males.^[11,12]^ In this study, we found that a loss-of-function BMPR2 mutation was associated with increased expression of AMHR2, which is phylogenetically close to BMPR2. Notably, AMH secreted by Sertoli cells of the testis is critical to male sex differentiation, inducing the regression of Müllerian ducts in the male fetus; it is also involved in testicular development and function.^[13]^ Males are born with higher AMH levels than females, to initiate sexual differentiation, and this tendency is maintained until puberty.^[14]^ On the other hand, females have very low serum levels of AMH by puberty when follicular development begins.^[15]^ In males, possible BMPR2 mutations are most likely compensated by the elevated expression of AMHR2, and symptomatic IPAH rarely develops. On the other hand, in females, the low levels of AMH early in life might associate with higher basal expression of AMHR2. Thus, the subsequent rise in serum AMH during adolescence might significantly increase AMH/AMHR2-dependent Smad signaling, resulting in symptomatic IPAH.

A recent study reported evidence for a complementary relationship between AMH/AMHR2 and BMPs/BMPR2 signaling pathways.^[16]^ In particular, AMH knockdown in pulmonary epithelial cell-lines specifically increased the expression of BMPR2, whereas AMH overexpression decreased BMPR2, but not TGF-βR2 expression.^[16]^ AMHR2, originally known to function in gonadal tissue, is also expressed in lung epithelial cells, where it exerts a pro-survival and proliferative role by influencing basal and BMP-dependent Smad signaling.^[16,17]^ This evidence, together with our results, suggests that, in IPAH, attenuated BMPR2 signaling can induce AMHR2 in pulmonary arterial smooth muscle and endothelial cells. This hypothesis would account for the gender difference and suggest a possible molecular mechanism for IPAH pathogenesis. However, further studies are needed to confirm the role of AMH/AMHR2 signaling in IPAH.

An additional novel finding of this study is the apparently paradoxical activation of Smad1/5/8, despite attenuated BMPR2 expression (Fig. 2). A widely accepted hypothesis to explain BMPR2 mutation-related IPAH is the loss of homeostasis between BMP and TGF-β signaling, normally antagonizing each other.^[18,19]^ The loss of BMPR2 function and the subsequent reduction in Smad1/5/8 activation are coupled to enhanced Smad2/3 activation by the TGF-β signaling pathway, which may be responsible for the proliferation of pulmonary arterial smooth muscle.^[6]^ Moreover, primary pulmonary arterial smooth muscle cells (PASMCs) with a mutation in the kinase domain of BMPR2 showed defective Smad1/5 activation.^[20]^ However, in the PBMCs of our IPAH patient, we found increased phosphorylation of both Smad1/5/8 and Smad2/3, which are the intracellular signaling molecules of BMP and TGF-β pathways, respectively. This conflict can be derived from the difference in the experimental cell source of PBMCs and PASMCs due to a context-dependent TGF-β-antagonizing function of BMPR2. However, it is worth noting the difference in the BMPR2 mutation site. As mentioned above, the mutation in our patient was located in BMPR2 exon 2, encoding the extracellular ligand-binding domain and; therefore, resulted in a truncated BMPR2 protein, unable to reach the cell surface.^[7]^ On the other hand, BMPR2 with mutations in the intracellular signaling domain may maintain cell surface expression, albeit in the absence of protein function.^[21]^ Recent evidence revealed that, based on the type of mutation, BMPR2 can either display a “wild-type-like” subcellular localization, on the plasma membrane and in the perinuclear area, or be abnormally distributed in the cytoplasm.^[21]^ This mutation site-related difference in cell surface expression may affect the expression of complementary BMP receptors such as AMHR2, ALK1, and ALK6. Our result implies the presence of at least 2 different molecular mechanisms of BMPR2-associated IPAH.

In conclusion, we report a novel frameshift mutation (c.117InsT, p.Y40fsX48) of the *BMPR2* gene identified in a 19-year-old IPAH patient. Despite BMPR2 loss-of-function, the phosphorylation of Smad1/5/8 and Smad2/3 was enhanced in the patient's PBMCs, which was associated with an increased expression of BMP-signaling target genes. In turn, the enhanced activation of BMP signaling was related to the increased expression of both type I BMPRs, that is, ALK1, ALK3, and ALK6 and the type II BMPR, AMHR2 (Fig. 3C). Although it would be still premature to postulate a direct pathogenic role of enhanced BMP signaling, our results may propose a novel hypothesis for the enhanced BMP signaling in IPAH.

## Author contributions

**Conceptualization:** Sun Ha Choi, Seungwoo Han.

**Data curation:** Sun Ha Choi, Youn-Kwan Jung, Ji-Ae Jang, Seungwoo Han.

**Formal analysis:** Sun Ha Choi, Youn-Kwan Jung, Ji-Ae Jang, Seungwoo Han.

**Funding acquisition:** Seungwoo Han.

**Investigation:** Youn-Kwan Jung, Seungwoo Han.

**Methodology:** Sun Ha Choi, Youn-Kwan Jung, Ji-Ae Jang, Seungwoo Han.

**Resources:** Sun Ha Choi, Seungwoo Han.

**Supervision:** Seungwoo Han.

**Validation:** Youn-Kwan Jung, Ji-Ae Jang, Seungwoo Han.

**Visualization:** Youn-Kwan Jung, Ji-Ae Jang, Seungwoo Han.

**Writing – original draft:** Sun Ha Choi, Seungwoo Han.

**Writing – review and editing:** Sun Ha Choi, Youn-Kwan Jung, Seungwoo Han.

## Supplementary Material

Supplemental Digital Content
