# Supplementary material for: Idiopathic pulmonary arterial hypertension associated with a novel frameshift mutation in the bone morphogenetic protein receptor II gene and enhanced bone morphogenetic protein signaling: A case report
Source: Medicine (Baltimore). 2019 Oct 18;98(42):e17594. doi: 10.1097/MD.0000000000017594 (PMC6824762; doi:10.1097/MD.0000000000017594)
Supplement: Supplemental Digital Content [file medi-98-e17594-s001.docx]

**Idiopathic pulmonary arterial hypertension associated with a novel frameshift mutation in the bone morphogenetic protein receptor type II gene: Implications for the enhanced BMP signaling**

Sunha Choi^1^*, Youn-Kwan Jung^2^*, Ji-Ae Jang^3^, Seungwoo Han^1^

^1^Department of Internal medicine, Kyungpook National University Hospital, Daegu, Republic of Korea

^2^Biomedical Research Institute, Gyeongsang National University Hospital, Jinju, Gyeongsangnam-do, Republic of Korea

^3^Laboratory for arthritis and bone biology, Fatima Research Institute, Daegu Fatima Hospital; Daegu, Republic of Korea

**Supplementary Methods**

Sequencing of BMPR2 DNA

All participants provided written informed consent before blood sampling and DNA analysis. Genomic DNA was extracted from whole blood of participants by genomic DNA extraction kit (Cosmo Genetech, Korea). The reference sequence of human BMPR2 gene was obtained from the NCBI database (NM_001204.6) and genomic sequence containing introns was from Human BLAT Search tool of UCSC Genomic Institute (http://genome.ucsc.edu/cgi-bin/hgBlat). All exons of the BMPR2 gene were amplified by polymerase chain reaction (PCR) using primer pairs corresponding to each exon (Supplementary Table 1). Sequencing was performed directly on PCR products after purification at qualified specialized facility (GeneAll, Korea).

Isolation and in vitro culture of human PBMCs

Peripheral blood mononuclear cells (PBMCs) isolated from whole blood by Ficoll density gradient method (FIcoll Histopaque, Sigma-Aldrich #10771) were cultured in complete α-MEM. RNA expression was analyzed after incubation for 6 hr without growth factor stimulation. For Western blot analysis, cells were stimulated with BMP2 (200 ng/ml), TGFβ (20 ng/ml), and PMA (100 nM) for 24 hr.

Real-time quantitative PCR

Total RNA was isolated from PBMCs using EasyBlue RNA extraction kit (iNtRON, Korea). For real-time qPCR, 25ng of cDNA used as template and 10ul of SYBR green master mixture (Applied Biosystems, Waltham, MA) were mixed with gene-specific primers (Supplementary Table 1) in a total volume of 20 ul for each reaction. All samples were run in triplicate and normalized to GAPDH. The calculation of the relative expression was performed using the 2^-∆∆^CT method. Statistical analysis of the data was performed by Student’s t-test using the GraphPad Prism® software (La Jolla, CA).

Western blot analysis

Cell lysates from cultured mature osteoclasts were separated by SDS-PAGE and transferred onto a PVDF membrane. After blocking with 5% skim milk, immunoblotting was performed using antibodies listed below: anti-BMPR2 (#6979), p-Smad1(Ser463/465)/Smad5 (Ser463/465) (#9511), p-Smad2 (Ser465/467)/Smad3 (Ser423/425) (#8828), p-Erk1/2 (Thr202/Tyr204) (#9101), p-p38 (Thr180/Tyr182) (#4631) and AMHR2 (#4518) from Cell Signaling technology (Danvers, MA), ACVRL1 (ALK1, #sc-1-1556) from Santa Cruz Biotechnology (Dallas, TX), BMPR1A (ALK3, #38-6000) from Invitrogen (Waltham, MA), and BMPR1B (ALK6, #NBP2-14889) from Novusbio (Centennial, CO). The membranes were washed with TBS-T and incubated with HRP-conjugated secondary antibody (Santa Cruz Biotechnology).

Infection of Raw264.7 cells or BMMs with recombinant lentivirus was conducted in thepresence of 8 mg/ml polybrene (Sigma–Aldrich, St. Louis, MO) for 16 h, infected cells were selected in 3.5 mg/ml puro-mycin for 3–5 days.

Infection of Raw264.7 cells or BMMs with recombinant lentivirus was conducted in the presence of 8 mg/ml polybrene (Sigma–Aldrich, St. Louis, MO) for 16 h, infected cells were selected in 3.5 mg/ml puro-mycin for 3–5 days

Supplementary table. PCR primers used for amplification of BMPR2 exons and real-time qPCR in this study

| Targets | Primer sequence (5' to 3') | | Length |
| --- | --- | --- | --- |
|  | Forward | Reverse |  |
| Exon 1 | AACTAGTTCTGACCCTCGCCCC | GGACGCATGGCGAAGGGCAA | 602 |
| Exon 2 | TAGCTTCGCAGAATCAAGAA | TGCCTTGTTTTACAAGATTT | 177 |
| Exon 3 | TAGGATGTTGGTCTCACATT | TACTGAGTGGTGTTGTGTCA | 177 |
| Exon 4 | TAGGTCCACCTCATTCXATTT | TACCTGTCAACATTCTGTAT | 177 |
| Exon 5 | TAGGAGACCGTYAAACAAGGT | TACCTCCAACAGTTTCAGAT | 98 |
| Exon 6 | CAGCTGATTGGCCGAGGTCG | TACATTGGGATAGTACTCCA | 237 |
| Exon 7 | TAGGGATCTTTATGCAAGTA | TACCTCCTCGTGGTAATTCT | 121 |
| Exon 8 | GCAGAAAAATAATACTACTTCTATA | GATGTTTTAATTAAATTATCATTTC | 319 |
| Exon 9 | AGAATATGCTACGTTCTCTC | ACACTAGATAGCAATGAACTAAAGG | 336 |
| Exon 10 | GTATCAGAAATACCCCTGTT | TTAGGCAACTCCAAAAACTAT | 328 |
| Exon 11 | GGTAAACTGAAAAGCTCAATAC | CATTGAACTATTAGGCTGGT | 345 |
| Exon 12-1 | GATCCCCTTTCTTTCTTTAAGC | CTGTTTAAGAGAGTGCTCCATG | 510 |
| Exon 12-2 | GAACCTCAAGGAAAGCTCTG | AGCATGGGAGTTAACACTGT | 436 |
| Exon 12-3 | ACCTCATGTGGTGACAGTCA | ATTGGAATTAGTTCGGCCAC | 316 |
| Exon 12-4 | ATTCCAGTCCTGATGAGCAT | AGTTATTTAAATGGCCCCAA | 343 |
| Exon 13 | TTACATCCCTTACCCGTTAT | TTAAAGCAAGTCTTTGTTGC | 454 |
| ID1 | CCAGAACCGCAAGGTGAG | GGTCCCTGATGTAGTCGATGA | 62 |
| SMAD6 | CCAGGAGAAACTCGCTCCAA | ATCCCTGGATTTGCATGCA | 80 |
| STAT1 | TGAGTTGATTTCTGTGTCTGAAGTT | ACACCTCGTCAAACTCCTCAG | 90 |
| ATF4 | GGTGGCCAAGCACTTCAAAC | CATCCACAGCCAGCCATTC | 80 |
| GADD45b | AGCGTGGTCCTCTGCCTCTT | TGGATGAGCGTGAAGTGGATT | 80 |
| EMP1 | CCAGTGAAGATGCCCTCAAGA | GAAGACCAGGAGGGCAATGA | 80 |
| MYC | AGAAATGTCCTGAGCAATCACC | AAGGTTGTGAGGTTGCATTTGA | 67 |
| ACVRL1 (ALK1) | CGGAGTTTCGCTCTTGTTGTC | TTTGAACCTGGGAGGTAGACGTT | 80 |
| ACVR1 (ALK2) | TGCGGTAATGAGGACCACTGT | CATAAACCTGGAAGCAGCCTTT | 100 |
| BMPR1A (ALK3) | ACCGAGTGATCCGTCATACGA | TCCACCGATTAGACACAATTGG | 70 |
| ACVR1B (ALK4) | CAGAACCTTGGCGGTTTATAGG | GCTTGGGAGTGGACGTGTGT | 100 |
| TGFBR1 (ALK5) | GCCCCTGAAGTTCTCGATGA | TGGAACATCGTCGAGCAATTT | 111 |
| BMPR1B (ALK6) | TTGGCCTCCTCAGACCACTT | TCCTAGACAGGCGGTGTTTGT | 90 |
| ACVR1C (ALK7) | CCCACTGCCATCACATCCA | TCTGGGAGCCAATTGCAGTT | 100 |
| ACVR2A | GCCCAGATCTCCGCGTAAG | GCTGTGCAGGGCATAGACTTC | 100 |
| ACVR2B | GCGATTTAATTGGCTGGTCTTG | TGAGCATTAGCGACTGGAAATG | 80 |
| TGFBR2 | ATCTCCAGTCCACGTTCACAAA | TCCAGGTAGGCAGTGGAAAGA | 100 |
| BMPR2 | TTCCTTTGCAAACCGTCAGAA | CGGGCAATGTTGTCATGTTC | 81 |
| AMHR2 | GGCCCTGCTACAGCGAAAG | TCCAGTCCCTGCCTGAGTCT | 80 |
| GAPDH | AGCCACATCGCTCAGACAC | GCCCAATACGACCAAATCC | 66 |

Infection of Raw264.7 cells or BMMs with recombinant lentivirus was conducted in the presence of 8 mg/ml polybrene (Sigma–Aldrich, St. Louis, MO) for 16 h, infected cells were selected in 3.5 mg/ml puro-mycin for 3–5 days.

Infection of Raw264.7 cells or BMMs with recombinant lentivirus was conducted in the presence of 8 mg/ml polybrene (Sigma–Aldrich, St. Louis, MO) for 16 h, infected cells were selected in 3.5 mg/ml puro-mycin for 3–5 days
